# Generation of recombinant mAbs to vaccinia virus displaying high affinity and potent neutralization

**DOI:** 10.1128/spectrum.01598-23

**Published:** 2023-09-22

**Authors:** Tal Noy-Porat, Hadas Tamir, Ron Alcalay, Ronit Rosenfeld, Eyal Epstein, Lilach Cherry, Hagit Achdout, Noam Erez, Boaz Politi, Yfat Yahalom-Ronen, Shay Weiss, Sharon Melamed, Tomer Israely, Ohad Mazor, Nir Paran, Efi Makdasi

**Affiliations:** 1 Israel Institute for Biological Research, Ness Ziona, Israel; Technion-Israel Institute of Technology, Haifa, Israel

**Keywords:** vaccinia virus, orthopoxviruses, neutralizing antibodies, VIG, Mpox

## Abstract

Members of the *Orthopoxvirus* genus can cause severe infections in humans. Global vaccination against smallpox, caused by the variola virus, resulted in the eradication of the disease in 1980. Shortly thereafter, vaccination was discontinued, and as a result, a large proportion of the current population is not protected against orthopoxviruses. The concerns that the variola virus or other engineered forms of poxviruses may re-emerge as bioweapons and the sporadic outbreaks of zoonotic members of the family, such as Mpox, which are becoming more frequent and prevalent, also emphasize the need for an effective treatment against orthopoxviruses. To date, the most effective way to prevent or control an orthopoxvirus outbreak is through vaccination. However, the traditional vaccinia-based vaccine may cause severe side effects. Vaccinia immune globulin was approved by the U.S. Food and Drug Administration (FDA) for the treatment of vaccine adverse reactions and was also used occasionally for the treatment of severe orthopoxvirus infections. However, this treatment carries many disadvantages and is also in short supply. Thus, a recombinant alternative is highly needed. In this study, two non-human primates were immunized with live vaccinia virus, producing a robust and diverse antibody response. A phage-display library was constructed based on the animal’s lymphatic organs, and a panel of neutralizing monoclonal antibodies (mAbs), recognizing diverse proteins of the vaccinia virus, was selected and characterized. These antibodies recognized both mature virion and enveloped virion forms of the virus and exhibited high affinity and potent *in vitro* neutralization capabilities. Furthermore, these monoclonal antibodies were able to neutralize Mpox 2018 and 2022 strains, suggesting a potential for cross-species protection. We suggest that a combination of these mAbs has the potential to serve as recombinant therapy both for vaccinia vaccine adverse reactions and for orthopoxvirus infections.

**Importance:**

In this manuscript, we report the isolation and characterization of several recombinant neutralizing monoclonal antibodies (mAbs) identified by screening a phage-display library constructed from lymphatic cells collected from immunized non-human primates. The antibodies target several different antigens of the vaccinia virus, covering both mature virion and extracellular enveloped virion forms of the virus. We document strong evidence indicating that they exhibit excellent affinity to their respective antigens and, most importantly, optimal *in vitro* neutralization of the virus, which exceeded that of vaccinia immune globulin. Furthermore, we present the ability of these novel isolated mAbs (as well as the sera collected from vaccinia-immunized animals) to neutralize two Mpox strains from the 2018 to 2022 outbreaks. We believe that these antibodies have the potential to be used for the treatment of vaccinia vaccine adverse reactions, for other orthopoxvirus infections, and in cases of unexpected bioterror scenarios.

## INTRODUCTION

Members of the *Orthopoxvirus* genus can cause severe infections in humans. Among these, the variola virus, the causative agent of smallpox, was responsible for millions of deaths throughout history (1, 2). Other members, such as monkeypox (Mpox), cowpox (CPXV), and vaccinia (VACV), exhibit sporadic occurrences worldwide, with a recent, comprehensive outbreak of Mpox responsible for over 80,000 cases as of January 2023 (according to the Centers for Disease Control and Prevention) (1). Outbreaks of different VACV strains have also increased in the last 20 y in different areas of the world. Many VACV occurrences were reported in South America, especially Brazil (3, 4), while buffalopox (considered a VACV sub-lineage) and CPXV infections were reported mainly in the Indian subcontinent and Europe, respectively (1, 5). Although smallpox was declared eradicated in 1980, following a worldwide vaccination campaign (6) (see also: https://www.who.int/health-topics/smallpox#tab=tab_1), it is still considered a major biological threat. Over the years, this threat was further enhanced due to the gradual decrease in the number of vaccinated individuals following the termination of vaccination in the 1970s to 1980s. In addition, recent advances in molecular biology suggest that *de novo* synthesis of variola is now relatively easy and accessible. To date, the only way to prevent or control a smallpox outbreak is through vaccination. However, the vaccinia-based vaccine may, on some occasions, cause severe adverse effects such as progressive vaccinia, eczema vaccinatum, and post-vaccinal encephalitis (4, 7). Moreover, occasional outbreaks of other orthopoxviruses, which are becoming more frequent and prevalent (3 – 5, 8), emphasize the need for an effective and available treatment against orthopoxviruses. The live VACV vaccine was shown to elicit cross-protective immunity against a broad range of poxviruses (1, 9, 10). Antibodies (Abs) are believed to play a major role in the protection and elimination of the virus. Neutralizing antibody titers were identified as a correlate of protection during the global vaccination campaign (11, 12), and data from animal studies or vaccination studies in humans showed that Abs are necessary for protection against lethal poxvirus infections (13, 14). Likewise, cell-mediated immunity was also found to be important in controlling the primary infection, and memory T-cells were found to persist for decades in vaccinated individuals (12, 15 – 17). Vaccinia immune globulin (VIG), prepared from the plasma of vaccinated human donors, is a polyclonal antibody preparation licensed for treatment of smallpox vaccine adverse reactions and can also be used therapeutically to treat severe infections caused by other orthopoxviruses (including Mpox) in case of an outbreak (10). VIG, however, suffers from many disadvantages, such as batch-to-batch variability, poor efficiency, and the potential to transmit blood-borne pathogens. But the most crucial and urgent problem is the limited availability of VIG preparations due to the reduction in the number of vaccinated donors (18). Recombinant monoclonal antibodies (mAbs) may offer an unlimited, highly efficient, defined, and reproducible alternative.

Orthopoxviruses are large DNA viruses, encoding over 200 proteins. In the host, the virus exists in two distinct forms: the intracellular mature virion (MV) and the extracellular enveloped virion (EV) (19). The MV form can be found inside infected cells and is mainly involved in infection between hosts, while the EVs mediate dissemination within the host (20, 21). The EVs are formed from MVs that have been wrapped by an additional membrane, derived from the trans-Golgi network or endosomal membrane (22, 23). Therefore, the outer membrane of each form displays a distinct set of proteins. Proteins H3, D8, and A27 on the MV membrane were shown to be crucial for virus attachment to cells, while proteins A33, A34, and B5 on the EV membrane play important roles in virion formation, infection, and intracellular trafficking (12, 24 – 26). All of these proteins were previously shown to elicit a neutralizing and protective antibody response (10, 24, 27 – 30). Accordingly, an efficient antibody-based treatment against poxviruses should preferably contain antibodies against both MV and EV virion forms (10, 15, 18).

The aim of this study was to develop a set of potent, high-affinity monoclonal antibodies that will efficiently neutralize both infectious forms of the vaccinia virus, thus offering a potentially recombinant alternative to the plasma-derived VIG used today.

## RESULTS

### Immunization and serum characterization

**FIG 1:**
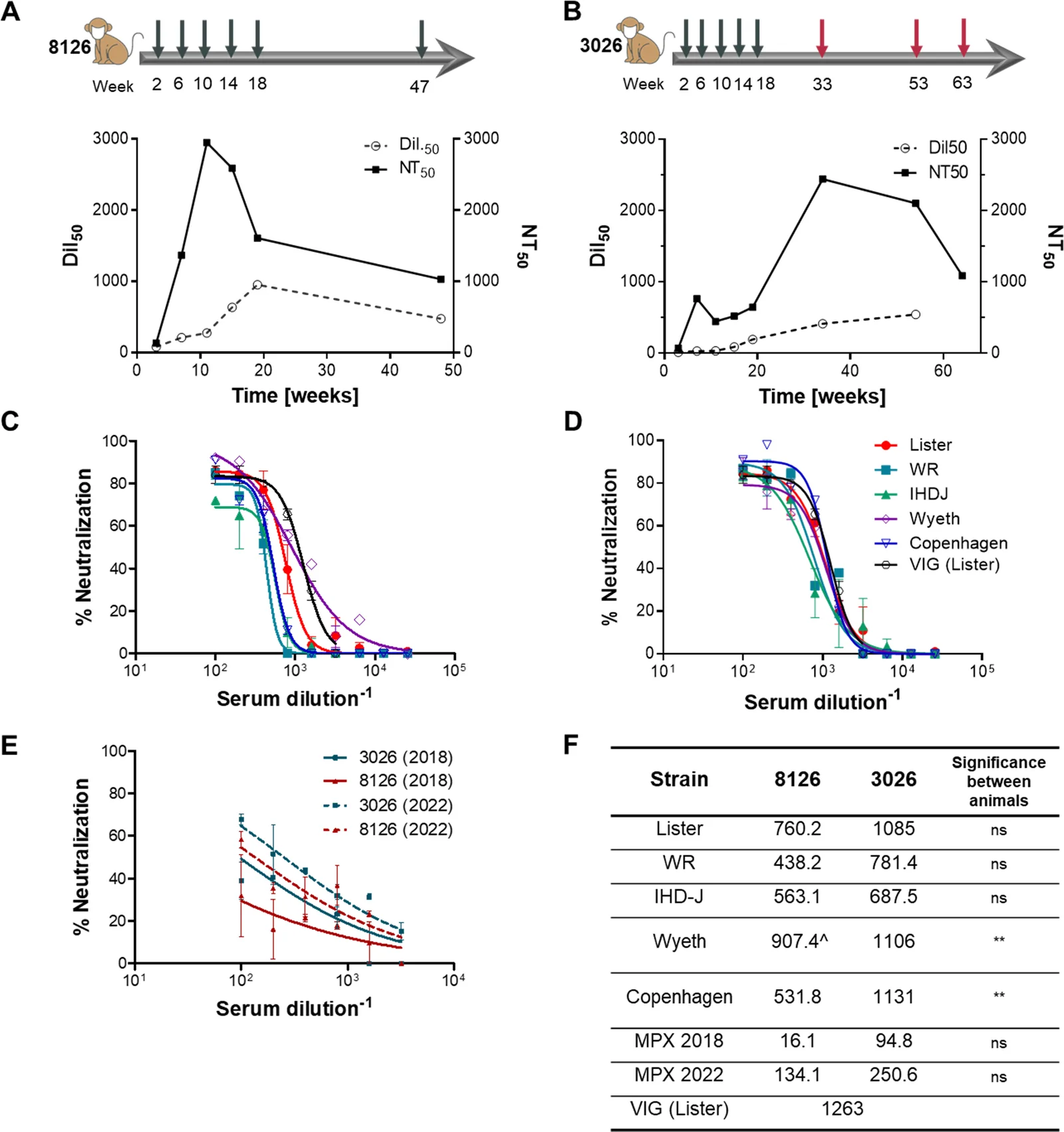
Immunization and serum characterization. Two NHP females were immunized with live VACV. Animal 8126 (**A**) received six doses of Lister strain (gray arrows), while animal 3026 (**B**) received five doses of Lister (gray arrows) and additional three boosts of WR strain (red arrows). The numbers on the bottom of each arrow indicate the time of each boost (in weeks from the beginning of immunization). Binding titer (Dil_50_) and neutralization titer (NT_50_) were measured 7 d after each boost, using ELISA against VACV IHD-J or neutralization assay against VACV Lister, accordingly. (C and D) Serum samples of animals 8126 (**C**) and 3026 (**D**), collected at the end point of immunization, were evaluated for their ability to neutralize different VACV strains, as indicated in the figure. A commercial VIG sample, tested on the Lister strain, was used as a control. (E) The same serum samples were tested for their ability to neutralize two Mpox strains, 2018 and 2022, using an *in vitro* MV neutralization test. (F) Summary of NT_50_ values resulting from neutralization tests described in (C–E). The significance between the neutralization curves of animals 8126 vs 3026 was tested using a one-way ANOVA on the AUC and Tukey’s post test. ns, not significant; **, *P* < 0.003; ^, significance compared to other strains in the same animal (*P* < 0.05). All data points represent the mean ± SEM of triplicates.

A prolonged and controlled immunization process against the VACV was carried out in order to evoke an effective immune response that would induce strong specific antibodies. Two non-human primates (NHPs; rhesus macaques) were subcutaneously immunized with 1 × 10^6^ PFU of live VACV, Lister strain, followed by four additional boosts of 1 × 10^9^ PFU each, given at monthly intervals. Antibody titer (Dil_50_) and neutralization potency (NT_50_) were monitored in blood samples along the course of immunization (Fig. 1A and B). Substantial increases in antibody titer and neutralization capabilities were observed for animal 8126 as of the first boost, with neutralization starting to decrease after the third immunization while binding titers continued to increase. Animal 8126 was given an extra boost 7 mo after the fifth immunization. Seven days later, the animal was sacrificed, and blood and lymphatic organs were collected for antibody library preparation. Animal 3026 showed only mild responses in both parameters throughout the first five boosts, probably due to individual differences in immunity. In order to try and expand the immune response, the animal received three extra boosts with the VACV Western Reserve (WR) strain, which originated from the New York City Board of Health strain and exhibits higher virulence in mice (31). Although not previously tested in primates, it was assumed that it may confer a stronger challenge in this model as well. This resulted in a marked increase in neutralization values and a more gradual but constant increase in antibody titers, eventually reaching similar values as in animal 8126 (Fig. 1B). Seven days after the last boost, the animal was sacrificed, and blood and lymphatic organs were collected for antibody library construction.

**FIG 2:**
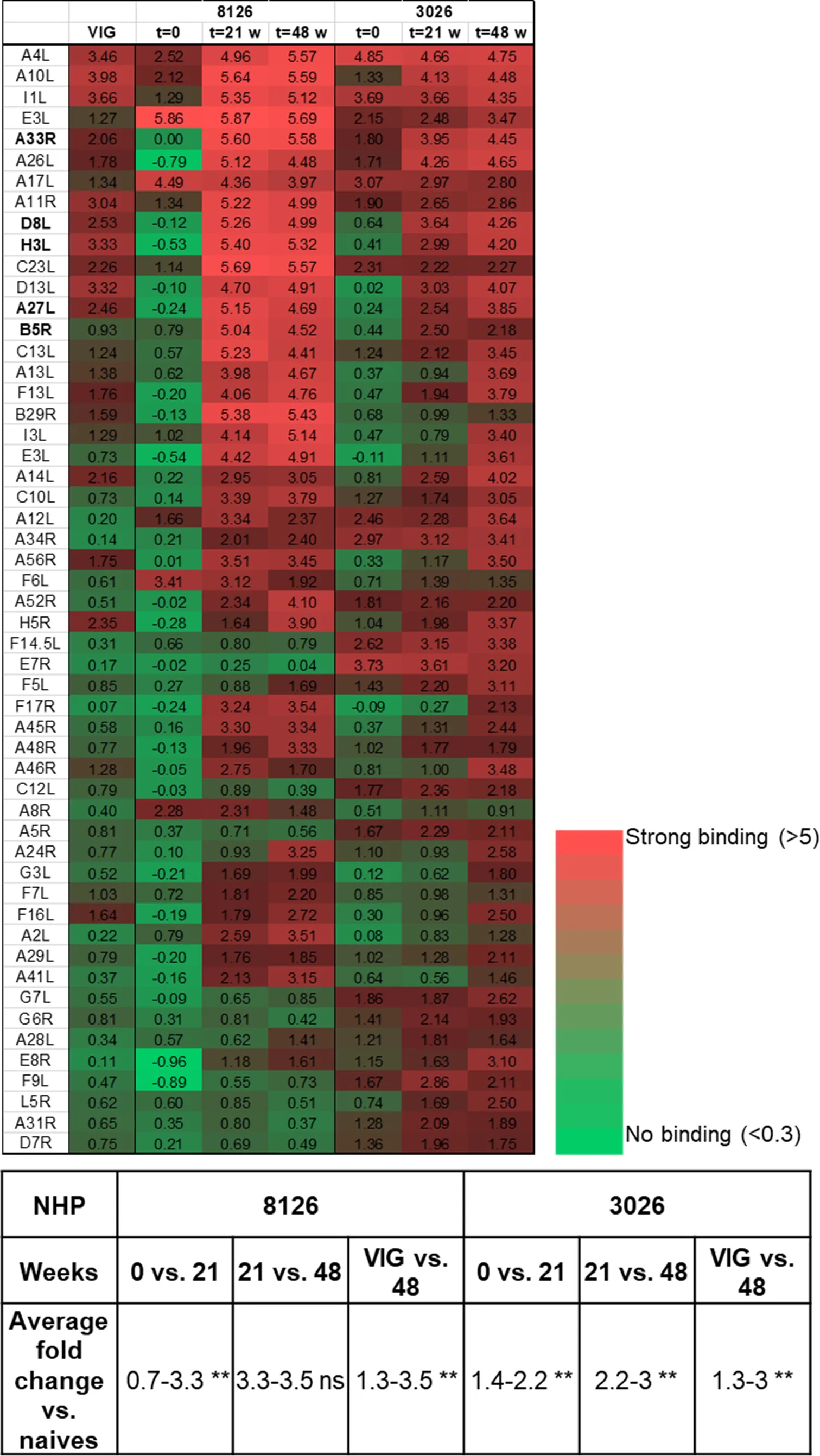
Antibody profile analysis of VACV-immunized NHPs’ sera. Serum samples taken from two animals, 8126 and 3026, before the onset of immunization and 21 and 48 wk after the onset of immunization (*t* = 0, *t* = 21, and *t* = 48, respectively), were analyzed against an array of 224 vaccinia proteins. Commercial VIG was used for comparison. Results obtained for the 50 strongest proteins are shown as a heat map according to the scale in the figure (numbers represent fold change vs naives). In bold are the five central membrane proteins known to elicit a protective immune response. Statistical analysis was performed between the different time points for both NHPs (0–21, 21–48 wk, and VIG compared to 48 wk) using a paired *t*-test. ns, not significant; **, *P* < 0.003.

In order to further evaluate the variability and potency of the immune reaction against VACV, the serum samples were analyzed to detect the presence of antibodies recognizing diverse proteins of the virus. Serum samples were collected before immunization (*t* = 0) and at two time points along the immunization process: at 21 and 48 wk after immunization initiation (*t* = 21 and *t* = 48). These samples were then analyzed using an array of 224 vaccinia proteins, compared to a commercial VIG (Omrix), as a reference (Fig. 2). Following immunization, a significant response could be detected against a diverse set of proteins, including the five surface proteins known to elicit a protective, neutralizing response: A33, D8, H3, A27, and B5. As could be expected, a comparable response was detected at weeks 21 and 48 for NHP 8126, probably because the immune reaction had already peaked after the fifth boost, administered at week 20. However, for NHP 3026, the additional boosts given with the WR strain clearly had a substantial impact, not only on antibody titers (see Fig. 1) but also on the diversity of the response (see Fig. 2). Thus, a marked and significant response against a larger number of proteins was clearly demonstrated for NHP 3026 at 48 wk compared to 21 wk. Importantly, not only was the whole repertoire of antibodies in the VIG preparation also represented in the NHP sera, but the extent of response observed for both primates’ sera was stronger and more diverse than that observed for the VIG sample (Fig. 2).

Next, we analyzed the neutralization potential of the antibodies in the animal’s serum at the endpoint of immunization. Since the two NHPs responded differently to the Lister strain, we wished to confirm that the antibodies developed following the entire immunization process will be able to neutralize different VACV strains. An *in vitro* neutralization test was carried out using several VACV strains, including Lister, WR, IHD-J, Wyeth, and Copenhagen. A similar neutralization potency (NT_50_ values of 450–900 for 8126 and 700–1,100 for 3026) was observed for all strains (Fig. 1C and D). No significant difference was observed between neutralization of the different strains by 3026 serum, while for 8126, neutralization of the Wyeth strain was significantly better than all the other strains (*P* < 0.05). Interestingly, serum 3026 could neutralize all of the VACV strains slightly better than serum 8126 (and this was significant for the Wyeth and Copenhagen strains; Fig. 1F), including the Lister strain, to which the initial immune response was very poor (Fig. 1B). This result suggests that the additional WR boosts given to animal 3026 resulted in an efficient and diverse immune response (as exhibited in Fig. 2) that eventually allowed improved protection against different VACV strains.

Mpox is another member of the orthopoxvirus genus and the causative agent of a disease that occurs from time to time, mainly on the African continent. In recent years, there have been two major Mpox outbreaks with identified cases outside of Africa: one in 2017–2019 and the second in May 2022. In light of the potential cross-reactive immunity between different orthopoxviruses, we sought to examine whether the antibodies in the immunized NHPs sera could neutralize the Mpox strains involved in these two outbreaks. Both animal sera were tested by *in vitro* neutralization assay against both Mpox strains (2018 and 2022; Fig. 1E). Both sera could neutralize Mpox with similar potencies, with serum 3026 exhibiting slightly better neutralization than serum 8126 (although these differences were not significant; Fig. 1E and F). Nevertheless, the neutralization of Mpox demonstrated by the two sera was inferior to that measured against VACV (Fig. 1F; *P* < 0.013 for 8126 and *P* < 0.008 for 3026 when compared to the Lister strain used for immunization). Interestingly, both sera neutralized the 2022 strain better than the 2018 strain (Fig. 1E and F; *P* = 0.02 for 3026, ns for 8126). This difference may suggest enhanced recognition of specific antigens of the 2022 strain or reduced virulence of this strain, as was recently suggested (32, 33).

The results of the NHPs’ sera analysis point to a strong, diverse, and neutralizing antibody response resulting from the hyperimmunization process. Therefore, it is expected to be a promising source for potent protective antibodies against various orthopoxvirus members.

### Antibody library construction and antibody isolation

Spleen, bone marrow, lymph nodes, and peripheral blood mononuclear cells (PBMCs) collected from both animals were used for RNA extraction, cDNA synthesis, and the construction of a phage display single-chain Fv (scFv) library, representing approximately 2 × 10^9^ distinct clones. In order to isolate diverse neutralizing antibodies against both the MV and the EV viral forms, three consecutive enrichment steps of panning were performed using several different antigens. Panning was first performed against the vaccinia IHD-J strain, which contains a point mutation in the A34 protein, resulting in enhanced EV formation and release, thereby exhibiting ~50% EV virions, compared to ~5% in other strains (34). Clones selected during this enrichment process were tested by ELISA for their ability to bind VACV IHD-J. An initial screen revealed that many of the antibodies isolated recognized the H3 protein, present on the surface of the MV form and known as an immunodominant protein. Since a more diverse set of antibodies was desired, the phage-display library was again used for panning, performed against recombinant A33 protein, a membrane protein of the EV form, and against VAC ΔH3 strain (35), a VACV strain lacking the H3 protein on its surface. All resulting VAC-specific binders from all panning procedures were expressed as full-length antibodies (in a scFv-Fc format) for further analysis.

### Antibody characterization

**FIG 3:**
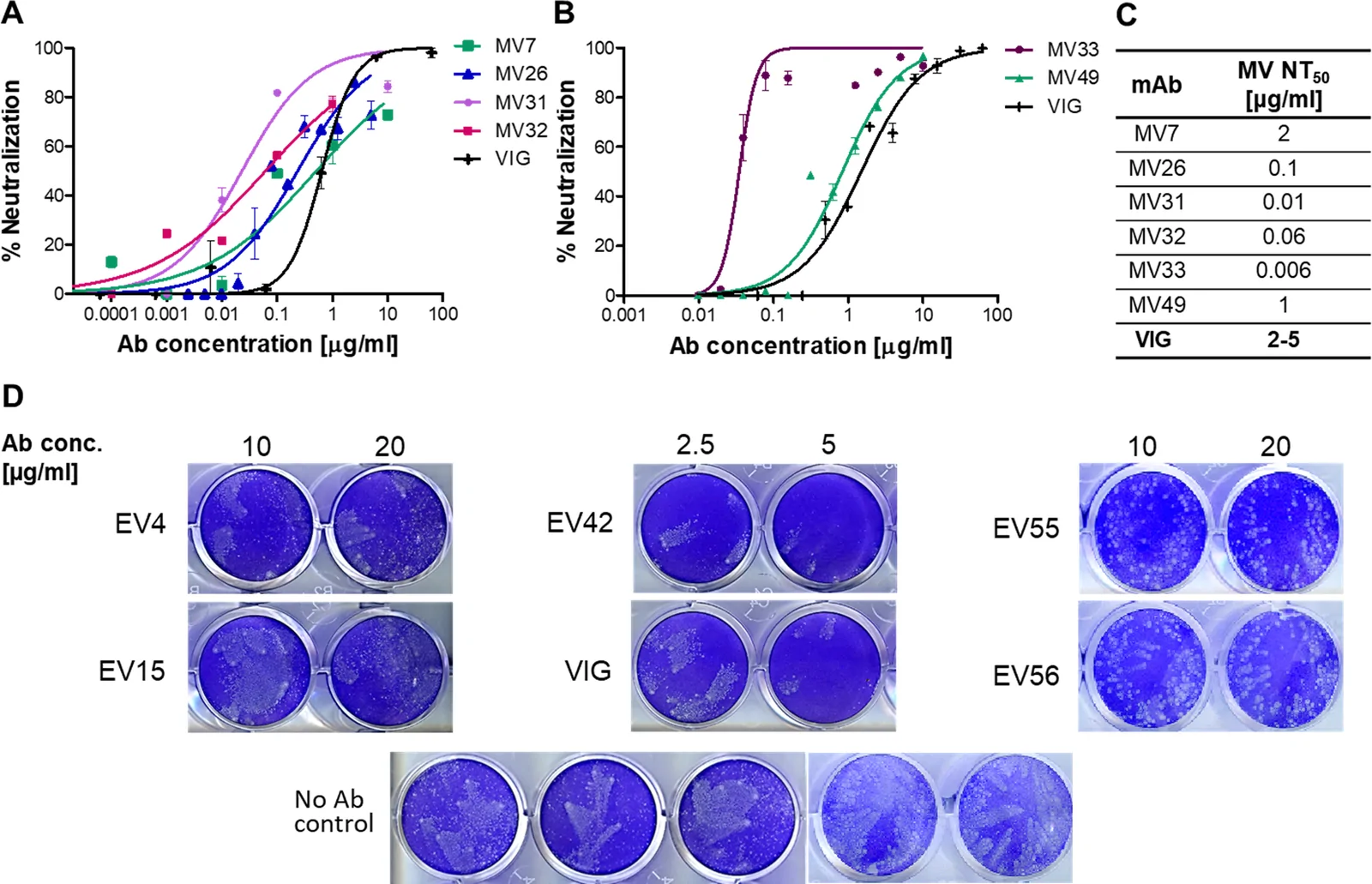
*In vitro* neutralization of monoclonal antibodies. Antibodies selected from the phage-display library were subjected to an *in vitro* MV neutralization assay using VACV-WR-vFIRE. The results of the most potent mAbs are presented in (A and B). All data points represent the mean ± SEM of triplicates. NT_50_ values obtained from this assay are summarized in (C). Antibodies that failed to exhibit MV neutralization were tested in an EV comet-inhibition assay. Antibodies presenting neutralization in this assay are presented in (D). Each mAb, indicated to the left of the wells, was tested at four concentrations (2.5–20 µg/mL), and the two lowest concentrations, exhibiting effective comet-inhibition, are presented (indicated at the top). A VIG sample was used as a reference in each MV and EV test, and a virus-only sample was used as a negative control. The assay was repeated twice for each mAb.

All mAbs were first subjected to an *in vitro* neutralization assay in order to characterize their neutralization potential and identify the form of the virus they recognize (MV or EV). First, mAbs were tested for their ability to neutralize MV virions using a high-throughput MV neutralization test. Only those mAbs that showed no neutralization were further examined in a comet EV neutralization assay. All neutralization assays included VIG samples for qualitative comparison, since any recombinant mAb that aims at replacing VIG must first display equal or improved potency. Figure 3A through D displays the results obtained for the most potent mAbs identified. Six mAbs exhibited effective MV neutralization capabilities that were superior to those exhibited by VIG, of which MV33 was found to be highly potent (Fig. 3B and C). Five mAbs were able to neutralize the EV form of the virus, with EV42 exhibiting the most effective neutralization (Fig. 3D). This mAb was able to block virus spread and comet formation even at a low antibody concentration (full inhibition at 5 µg/mL and partial but substantial inhibition at 2.5 µg/mL) and was more effective than VIG.

**FIG 4:**
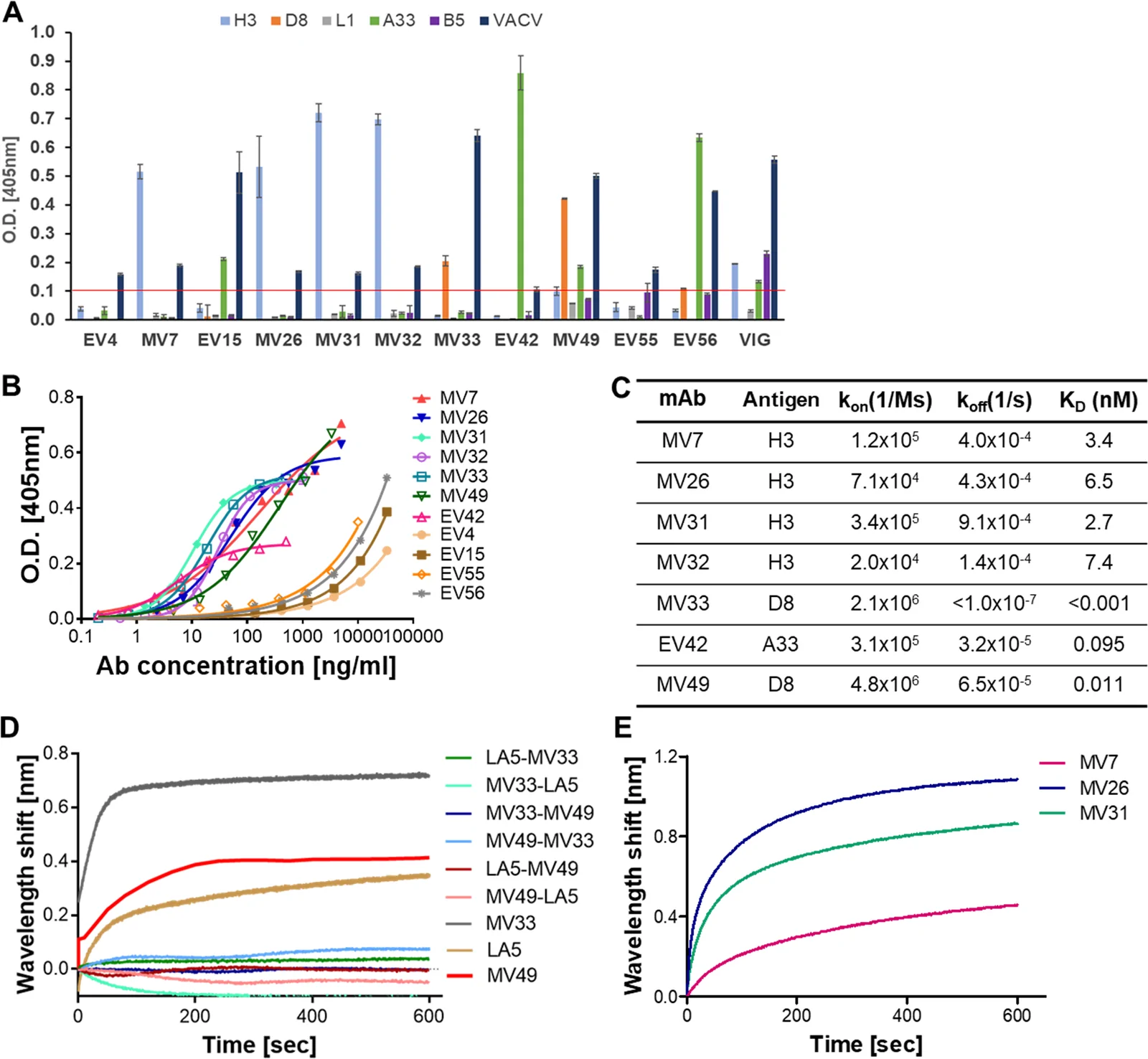
Binding characteristics of selected antibodies. (A) The specificity of the selected antibodies was determined by ELISA against the indicated proteins and against VACV IHD-J as a control. A VIG sample was used as a positive control. Data represent the mean ± SEM of triplicates. Results above the threshold of 0.1 O.D. (represented by a red line) were considered positive. (B) The binding profile of antibodies was determined by ELISA using VACV IHD-J as the coating antigen. Data represent the average of triplicates ± SEM. (C) Binding characteristics of the antibodies were determined using BLI. All antigens carrying a His tag were immobilized to Ni-NTA sensors and interacted with increasing amounts of the relevant antibody. Binding kinetics were fitted using a 1:1 binding model. (D and E) Epitope binning experiments were conducted using BLI. The antigen was immobilized on the Ni-NTA sensor and saturated with the first antibody. The complex was then incubated with each of the indicated antibodies. Time 0 represents the binding to the mAb1-antigen complex. Binding was evaluated by the ability of each pair of antibodies to simultaneously bind the antigen. (D) Binding of mAbs to the D8 protein. Each pair of antibodies, indicated in the legend, was tested separately and is indicated according to the order of binding. A curve showing each individual mAb binding to D8 without competition is shown for comparison. (**E**) Binding of mAbs to H3 protein. Each of the indicated mAbs was allowed to bind first, and the graph represents the binding of MV32 to the mAb1-H3 complex.

In order to identify the targets of these mAbs, they were examined by specificity-ELISA against five major membrane proteins known to elicit a protective immunological response (Fig. 4A). Based on this assay, four mAbs were found to bind H3 protein: MV7, MV26, MV31, and MV32; two antibodies were found to bind D8 protein: MV33 and MV49; and additional three antibodies were characterized as anti-A33 protein: EV15, EV42, and EV56. Antibodies EV4 and EV55 could not bind any of the proteins examined and therefore probably recognize a different epitope on the EV form (Fig. 4A).

The affinity of the mAbs toward the whole virus was assessed next. The binding profile of all mAbs was obtained by ELISA against VACV IHD-J (Fig. 4B). Based on their binding profiles, the mAbs could be divided into two groups, with one group, consisting of mAbs EV4, EV15, EV55, and EV56, exhibiting a distinct binding profile, reflecting relatively low binding affinity toward the virus compared to the second group. Interestingly, all of these mAbs recognize the EV form of the virus. Nevertheless, EV42, which also binds the EV form, displayed substantially stronger binding to the virus, although it demonstrated a lower Bmax value than the other mAbs in this group, which probably reflects fewer available epitopes. The remaining mAbs showed similar binding profiles toward the virus.

K_D_ values were determined for the antibodies exhibiting stronger binding using biolayer interferometry (BLI; Fig. 4C). MV33, recognizing the D8 protein (Fig. 4A), was found to have an excellent affinity toward the protein. The association rate of this antibody was fast, 2.1 × 10^6^, while the dissociation rate was extremely slow (below the Octet^Red^ detection limit of 1 × 10^−7^ s^−1^) and hence could not be measured. This puts the affinity of the mAb in the sub-pM range (k_off_ <1 × 10^−7^ s^−1^). MV49 also exhibited an extremely high affinity toward D8 (0.011 nM) despite its inferior neutralization efficiency compared to MV33 (Fig. 3B). EV42 was found to recognize the A33 protein with a very high affinity (0.095 nM), while all four mAbs recognizing the H3 protein exhibited similar affinities toward the protein in the low nM range (2.7–7.4 nM). Despite their comparable affinities, the anti-H3 mAbs differed in their ability to neutralize the virus, with MV31 and MV32 showing strong neutralization, MV26 exhibiting an order of magnitude lower neutralization potency, and MV7 showing even lower neutralization capabilities (Fig. 3A and C). These differences may suggest that the mAbs bind different epitopes on the H3 protein, with some epitopes allowing more efficient neutralization than others.

In an attempt to further characterize the mAbs specific recognition sites, binning experiments were conducted using BLI. In order to try and elucidate the MV33 epitope, we conducted a binning experiment against the antibody LA5. This murine antibody was previously shown to target the D8 protein, and its epitope was identified (36). Binning results showed that LA5 could not bind in the presence of MV33 (Fig. 4D); however, when LA5 was first allowed to bind D8, mAb MV33 was able to bind the protein to a minor extent. This may suggest a partial overlapping epitope, allowing a low level of binding of MV33 owing to its very high affinity. Next, we examined whether MV33 and MV49 mAbs can bind the D8 protein simultaneously. Again, MV49 was unable to bind D8 in the presence of MV33, but MV33 exhibited residual binding when MV49 was allowed to bind first (Fig. 4D). MV49 and LA5 also could not bind the D8 protein at the same time. The comparable binding characteristics of MV33 and MV49, in contrast to their different neutralization capabilities, may reflect partially overlapping epitopes or steric interference in joint binding.

Similarly, the ability of the four anti-H3 mAbs to bind the protein simultaneously was tested using BLI. MV32 was able to bind the H3 protein in the presence of all other mAbs (Fig. 4E), while the other mAbs could not bind simultaneously, implying that MV32 recognized a unique epitope on the H3 protein. However, when MV32 was allowed to bind first, none of the other mAbs could bind the protein (data not shown). This may suggest that all mAbs share adjacent epitopes, with MV32 presenting more pronounced steric interference than the others. Alternatively, this may imply that upon binding, MV32 induces a conformational change of the H3 protein, thus preventing the binding of other antibodies to the protein.

Three antibodies were recognized as binders of the A33 protein of the EV form; however, since A33 is a relatively small protein and presents a small surface area, antibodies that bind this protein often cross-block each other (37); therefore, binning experiments on A33 are not informative for epitope identification.

**FIG 5:**
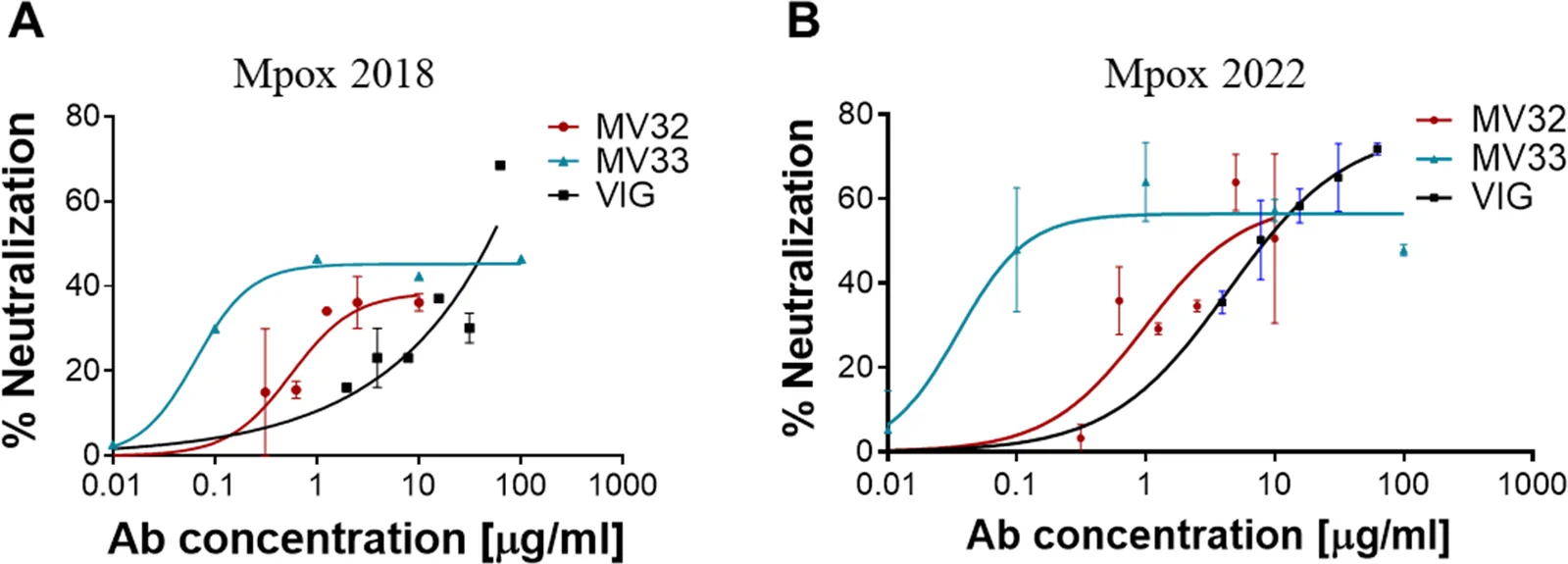
Mpox *in vitro* neutralization. Representative mAbs, MV32 and MV33, were tested for their ability to neutralize Mpox strains 2018 (**A**) and 2022 (**B**) in an *in vitro* MV neutralization assay. VIG samples were used as controls. Data represent the mean ± SEM of duplicates.

Given the notable binding and neutralization capabilities of the isolated mAbs against VACV and the sera’s capability to neutralize Mpox, we next sought to examine whether these mAbs can also neutralize the Mpox virus. MV33 mAb was selected as a representative of the antibodies recognizing D8 protein and MV32 as a representative of the mAbs binding H3. Both were tested in an *in vitro* MV neutralization assay against both Mpox 2018 and Mpox 2022 strains (Fig. 5). Both antibodies exhibited very efficient neutralization of the two Mpox strains with similar NT_50_ values (0.5–0.99 and 0.03–0.06 µg/mL for MV32 and MV33, respectively). However, the maximum neutralization percentage exhibited by both mAbs reached only around 60%. This may suggest a slightly reduced neutralization efficiency compared to VACV or the presence of EV particles in the virus samples. Nevertheless, both mAbs neutralized Mpox better than VIG (Fig. 5), with MV33 showing significantly better neutralization than VIG of both strains (*P* = 0.01 and *P* = 0.0003 for the 2018 and 2022 strains, respectively) and MV32 exhibiting significantly better neutralization of the 2022 strain (*P* = 0.005). Similar to the NHP serum, the VIG sample also exhibited a marked and significant reduction in neutralization (*P* < 0.0001) of Mpox compared to VACV. We could not accurately assess the Mpox neutralization potential of EV42, which binds the A33 protein and neutralizes the EV form of the virus, since the comet-inhibition assay is not currently adapted for Mpox.

## DISCUSSION

In the present study, we aimed to isolate specific and potent antibodies from two NHPs, using several different antigens for selection. A set of high-affinity and potent mAbs recognizing different epitopes of the virus was consequently isolated.

The two immunized NHPs displayed a marked difference in the response toward the immunization strain (VACV Lister), with animal 8126 developing a substantial immune response as of the first boost and animal 3026 showing a very weak response, even after five boosts. However, administering three boosts of the VACV WR strain to this animal resulted in a pronounced increase in antibody titers, eventually leading to an efficient antibody response in terms of diversity (Fig. 2) as well as neutralization efficiency against diverse strains and species (Fig. 1C through F).

It seems that a large proportion of the neutralizing response against the MV form conferred by the immunization process was directed against the H3 protein. Indeed, this protein was previously shown to be an immunodominant antigen in both humans and animals vaccinated with VACV, and anti-H3 antibodies were shown to be effective *in vitro* and protective in animal models (30, 38, 39). In this study, we have identified several neutralizing mAbs targeting the H3 protein, with MV31 and MV32 displaying especially efficient neutralization, with NT_50_ values greatly exceeding those of commercial VIG (Fig. 3A and C). MV32 was also found to efficiently neutralize Mpox. An attempt to bypass the immune response against H3 by using a ΔH3 VACV strain resulted in the isolation of antibodies against several other proteins, but these were less efficient neutralizers. It can therefore be assumed that the neutralizing immune response induced in the animals following immunization was considerably based on anti-H3 antibodies, as was seen in other studies that monitored the antibody response following vaccination (15, 39). It should be noted that a large number of antibodies were isolated, but only those showing neutralization potential were further characterized. Therefore, the non-neutralizing antibodies may have targeted other diverse proteins of the virus.

Despite the obvious dominance of the response against the H3 protein, the most potent antibody, MV33, was directed against the D8 protein, another central membrane protein of the MV form responsible for binding to host cells (40). This antibody exhibited exceptional affinity toward the D8 protein as well as excellent *in vitro* neutralization capability, with an NT_50_ value of 0.006 µg/mL (Fig. 3B and C). Additionally, this antibody exhibited potent Mpox neutralization (Fig. 5). Based on the high homology between the D8 proteins among the orthopox members, it seems that this mAb has the potential to recognize and neutralize additional ortopoxviruses. Interestingly, the maximal Mpox neutralization observed for both MV32 and MV33 was approximately 60%, compared to 80%–100% for VACV strains. Such a phenomenon was reported before (10) and may result from the presence of EV virions in the virus sample or from variations in virus preparations. Another mAb found to recognize the D8 protein is MV49, which also exhibited an excellent affinity toward the protein but was much less efficient in neutralizing the virus *in vitro* (Fig. 3B and C). The D8 protein is involved in the infection of cells by MV virions through its binding to the chondroitin sulfate (CS-E) receptor. Studies have shown that the binding site of the protein to the CS-E receptor is shaped as a positively charged crevice (29). Previously, antibodies recognizing the D8 protein that were found to be strong neutralizers were shown to bind the protein in the crevice area and completely block its binding to the CS-E receptor on cells (29, 37, 41). Since MV33 was found to be a very potent neutralizer *in vitro*, it may suggest binding to an epitope that effectively blocks D8 binding to its target receptor. Indeed, binning results showed that MV33 mAb apparently binds the same epitope of D8 as antibody LA5 (Fig. 4D), previously shown to bind D8 in the crevice area and block its binding to the CS-E receptor on cells. MV49 mAb was also unable to bind D8 in the presence of MV33, suggesting some degree of overlap between the two mAb epitopes. However, since MV49 exhibited inferior neutralization efficiency, it is possible that it only partially blocks the CS-E binding site.

Antibodies against the EV form of the virus are considered crucial for effective treatment and protection against orthopoxviruses (10, 24, 27, 42, 43). Nevertheless, the very low percentage of EV virions in any virus sample, their fragility and instability, and the fact that EV virions are considered less sensitive to neutralizing antibodies and to the complement cascade (19) make their isolation and development more complicated. Indeed, panning and selection of the phage-display library against the whole virus resulted in low numbers of anti-EV antibodies, which were all classified as poor neutralizers. However, selection with recombinant A33 protein resulted in the isolation of strong neutralizing antibodies. The A33 protein is a 21-kDa type II glycoprotein with a c-type lectin-like domain (44). It is required for efficient cell binding by EV particles, serves as a chaperon for other EV proteins, and is involved in the production of actin tails, thus making it essential for effective cell-to-cell spread within the host (26, 37, 45 – 48). Interestingly, most of the anti-EV antibodies isolated from the antibody library using any of the antigens were directed toward the A33 protein. This protein constitutes one of two surface proteins of the EV form known to elicit a protective response. The other major surface protein is B5, which has been shown to elicit neutralizing and protective antibodies in other studies (18, 27, 49). Among the anti-EV antibodies, EV42 mAb was prominent in its excellent affinity toward the virus and efficient *in vitro* neutralization, and it also exhibited the neutralization potential of Mpox, suggesting that it is a potent cross-species neutralizing antibody.

The NHP sera and several mAbs were evaluated for their ability to neutralize Mpox strains 2018 and 2022. Mpox was previously classified into two clades, with clade I originating in Central Africa (previously known as the Congo Basin clade) and clade II originating from West Africa (50). In recent years, two major Mpox outbreaks have occurred: one during 2017–2019, in which over 300 cases were identified, several of them outside of Africa (51), and the second is the current 2022 outbreak, in which the chain of transmission originated in Africa but spread to other non-endemic countries, mostly in Europe and the Americas. Both of these strains are more closely related to the West African clade. However, lately it has been proposed that isolates from these two outbreaks should be classified into a different clade based on their distinct geographical distribution and genomic and epidemiological differences (52, 53). Data on the genomic epidemiology of monkeypox virus from recent years can be found at Nextstrain.org/monkeypox/hmpxv1. The 2022 outbreak is caused by lineage B.1 of clade III, according to this classification (54, 55). Comparative genomic analysis revealed 46 single-nucleotide polymorphisms specific for this lineage, which reflect a higher than expected rate of genomic variance, suggesting accelerated evolution among the outbreak sequences (52, 55). This phenomenon was suggested to be related to the different geographic and demographic distributions of this outbreak compared to previous ones (53). The overall genetic similarity observed for the 2022 strain compared to VACV (GenBank accession no. NC_006998.1) is 84% (56). This may explain the difference in neutralization potency exhibited by the animal’s sera and VIG toward VACV compared to Mpox (Fig. 1C through E; Fig. 5). Interestingly, NT_50_ values measured for the 2018 strain were substantially lower than values measured for VACV Lister, while the NT_50_ values measured for the 2022 strain declined less dramatically (Fig. 1E and F), suggesting that the additional mutations observed in the 2022 strain did not damage and maybe even strengthened the cross-reactive capability of the antibodies produced following VACV immunization. Recently, it was suggested that the 2022 strain (lineage B.1) is evolving diminished virulence (32), which may explain our observation.

According to several studies, some of the differences observed in clade III are associated with coding regions of immunogenic proteins such as H3L and B21R (53, 55); however, the H3 protein of Mpox still exhibits 94% similarity to the VACV ortholog (56). Accordingly, mAb MV32, found to target H3, retained its high neutralization potential toward VACV and was able to efficiently neutralize Mpox *in vitro* (Fig. 5). Similarly, the D8L ortholog of Mpox also demonstrates 94% homology to its VACV counterpart, again explaining the high neutralization efficiency of MV33 against Mpox. Gilchuk et al. (10) previously reported that antibodies targeting VACV proteins H3 and A33 present the broadest cross-neutralizing activity and are able to neutralize Mpox and CPXV. However, in their study, anti-D8 mAbs were able to bind Mpox but were unable to neutralize it, in contrast to our observation. We assume that this difference is related to the superior affinity of MV33 for targeting D8, which probably accounts for its excellent neutralization capabilities. The observed differences between the ability of the monoclonal antibodies isolated against VACV to neutralize Mpox and the lesser efficiency of the animal serum can therefore be explained by the high homology between the important surface proteins shared by both species compared to the somewhat lower homology between the whole genomes of both species, which may point to the importance of developing specific monoclonal antibodies to treat orthopoxviruses rather than polyclonal preparations.

Several studies have previously reported the isolation of mAbs targeting different antigens of VACV. MAbs against H3 and B5 proteins were isolated from transgenic KM mice (49); mAbs targeting B5 and A33 were isolated from chimpanzee origin (27, 42); and several studies reported the isolation of diverse human mAbs from vaccinated donors using hybridomas, single-cell, and phage-display technologies (10, 18, 57 – 59). Most mAbs isolated target the same surface proteins mentioned before, emphasizing their importance in viral infection and spread. All of these mAbs were shown to be effective in neutralizing the virus; nevertheless, a recombinant treatment is not yet available, perhaps due to financial reasons derived from the need to manufacture a combination of several mAbs. However, the re-occurring and spreading outbreaks of orthopox viruses in recent years should give renewed motivation for the development of a recombinant therapy. In this study, NHPs were immunized with the VACV Lister strain, unlike previous studies in which other strains were used for immunization. This may contribute to the broadening of the existing antibody repertoire. Furthermore, the antibodies we reported here display higher affinities and better NT_50_ values than most mAbs previously reported. The affinities reported here ranged from the low nM to sub-pM range, while most antibodies reported exhibited affinity in the µM to high pM range. Accordingly, NT_50_ values that were reported ranged from 0.02 to 100 µg/mL (10, 18, 27, 42, 57, 58, 60), compared to the mAbs reported here, which display NT_50_ values of 0.006 to 2 µg/mL. We therefore believe that these mAbs could potentially provide excellent protection against orthopoxviruses.

To conclude, we report here the development of several high-affinity, potent neutralizing mAbs against VACV that recognize three different surface proteins present on both forms of the virus and also exhibit the neutralization potential of Mpox. Two NHPs were used for the immunization process, and slightly different immunization regimes were carried out for each one of them. This resulted in a strong and diverse immune response, which aided in the isolation of potent mAbs. As only two NHPs were vaccinated differently to generate a diverse repertoire of antibodies, the observed differences in their immunization remain qualitative. The exact efficiency of the mAbs isolated will have to be evaluated by *in vivo* experiments. Nevertheless, an antibody therapy containing these mAbs has the potential to treat smallpox vaccine adverse reactions and may also be examined as a therapy for other emerging orthopoxviruses.

## MATERIALS AND METHODS

### Viruses and proteins

VACV Lister (Israeli Ministry of Health) was grown on the chorioallantoic membranes (CAMs) of 12 d SPF embryonated eggs according to the protocol for vaccine production of the Israeli Ministry of Health (61). The virus was titrated on Vero (ATCC-CCL-81) cells. The CAMs were ground in glycerol buffer, filtered through a stainless-steel filter, and frozen. This strain was used for NHP immunization as well as neutralization assays.

VACV-WR used for immunization was propagated in HeLa (ATCC-CCL-2) cells, prepared as crude, and titrated on BSC-1 cells (ATCC-CCL-26).

VACV IHD-J was grown in HeLa cells and titer determined in BSC1 cells. For commet-inhibition assays, crude viruses were used. For ELISA and panning, the virus was purified by centrifugation in sucrose density gradient (using sucrose cushion) and inactivated using βpL.

The following recombinant vaccinia viruses were obtained from Bernard Moss (NIH): VACV-WR-vFire, VACV-IHDJ-vFire, VACV-Wyeth-vFire, and VACV-Copenhagen-vFire, expressing both GFP and Firefly luciferase under the control of an early-late promotor (62). Viruses were grown in HeLa cells and titer determined in Vero cells.

VACV ΔH3 was obtained from B. Moss (NIH), grown in BSC1 cells, and inactivated using βpL.

MV preparations-VACV strains were propagated in HeLa cells in Dulbecco’s modified Eagle’s medium (DMEM) supplemented with 10% fetal calf serum, 1% glutamine, 1% non-essential amino acids (NEAAs), and 0.5% Pen Strep Nistatin in T175 flasks. Cells were infected at a multiplicity of infection (MOI) of 0.5 and harvested 3 d post infection. The virus was recovered by rapidly freeze-thawing the cell pellet three times and sonicating for 1.5 min on ice. Cell debris was removed by centrifugation (at 1,200 rpm for 10 min), and the virus was aliquoted and stored at −80°C.

Monkeypox viruses 2018 (Mpox2018; accession no. MN648051) and 2022 (Mpox2022; accession no. ON649879) were obtained from clinical samples monitored during the mentioned outbreaks. The surveillance of monkeypox cases, collection, and analysis of the data were executed by health authorities and the Israel Institute for Biological Research as a national reference laboratory for identification and characterization of Mpox according to their mandate provided by the Israeli Ministry of Health. All samples were processed in an anonymized fashion. Viruses were grown in HeLa cells and prepared as described above (“MV preparations”). Titer was determined in BSC1 cells (63, 64). The final preparation was not further purified as previously described.

Expression of VACV A33 recombinant protein - A33R gene sequence, corresponding to amino acids 89–185 of A33 protein, was cloned to pRSET vector and expressed in *Escherichia coli*. N-terminal his-tag was included in the construct in order to facilitate protein purification. Recombinant proteins: H3, A27, L1, A33 and B5 were purchased from Sino Biological (Beijing, China). D8 protein was purchased from Cusabio Technology (Houston, TX, USA). The characteristics and source of the recombinant proteins used are listed in Table 1.

**TABLE 1:** Recombinant proteins

Protein	Source	Expression host	Sequence	Cat#	Tag
H3	Sino Biological	HEK293 cells	N/A	40893-V08H1-100	C ter-His
A27	Sino Biological	*E. coli*	Met1-Glu110	40897-V07E-100	N ter-His
L1	Sino Biological	HEK293 cells	Ala3-Gly183	40903-V07H-100	N ter-His
A33	Sino Biological	*E. coli*	Val57-Asn185	40896-V07E-100	N ter-His
A33	In house prep.	*E. coli*	aa89-185	N/A	C ter-His
B5	Sino Biological	HEK293 cells	Tyr18-His279	40900-V08H-100	C ter-His
D8	Cusabio Technology	*E. coli*	Met1-Asn304	CSB-EP322653VAA	N ter-His

LA5 antibody was obtained from Dirk Zajong (La Jolla Institute).

### Animal immunization

Two non-human female primates (*Macaca mulatta*) were immunized with live VACV Lister, 1 × 10^6^ PFU per animal, followed by four monthly booster injections of 1 × 10^9^ PFU. The first animal (8126) received a fifth booster injection of VACV Lister 7 mo after the last boost (48 wk from the onset of immunization) and 7 d later was sacrificed, and samples were taken from the blood, spleen, bone marrow, and lymphatic nodes. The second animal (3026) received three additional booster injections of the VACV WR strain: 33, 53, and 63 wk from the onset of immunization. Seven days after the last boost, the animal was sacrificed, and samples were taken from the blood, spleen, bone marrow, and lymphatic nodes.

Treatment of animals was in accordance with regulations outlined in the U.S. Department of Agriculture’s Animal Welfare Act and the conditions specified in the Guide for Care and Use of Laboratory Animals (National Institute of Health, 2011). Animal studies were approved by the local ethics committee on animal experiments (protocol number RM-01-2015).

### Phage display scFv library construction and panning

Total RNA was purified from all organs sampled using the RNeasy mini kit (Qiagen GmbH, Germany). CDNA synthesis was performed using the Verso cDNA synthesis kit (Thermo Scientific, USA) and used as a template for Abs variable region coding fragment amplification. Briefly, heavy and light Ig variable domains (V_H_ and V_L_) were amplified using a specific primer set (65). The V_H_ and V_L_ used in the PCR overlap extension reaction resulted in the scFv repertoire being cloned into the pCC16 phagemid vector (66) using *Nco*I/*Not*I. A total of 2 × 10^9^ independent clones were obtained, representing the library’s complexity.

For phage production, 25 mL of logarithmic bacteria culture (OD_600_ = 0.6) in YPD supplemented with 100 µg/mL ampicillin and 1% glucose (YPD-Amp-Glu) was infected with M13KO7 helper phage (New England Biolabs, USA) at 7 × 10^9^ plaque-forming unit (PFU) per mL (~1:20 multiplicity of infection) by incubating at 37°C for 30 min without shaking, followed by 30 min at 120 rpm. Infected cells were harvested by centrifugation (1,800 × *g* for 5 min) and resuspended in 100 mL YPD supplemented with 100 µg/mL ampicillin and 50 µg/mL kanamycin. After overnight growth at 30°C at 200 rpm, the cells were removed by centrifugation (1,800 × *g* at 4°C for 10 min). The culture supernatant containing the phages was filtered through a 0.45-µm filter and then precipitated with 1/5 vol of 20% PEG6000 (polyethylene glycol) in a 2.5 M NaCl solution for 2 h on ice. Phage particles were pelleted by centrifugation (9,000 × *g* at 4°C for 1 h) and re-dissolved in 5 mL of Dulbecco’s phosphate buffered saline (PBS; Biological Industries, Israel).

Panning was performed against inactivated VACV, IHD-J or ΔH3 strains, or recombinant A33 protein, directly absorbed onto polystyrene plates (Maxisorp 96-well microtiter plates; Nunc, Sigma-Aldrich, USA), and against biotinylated-VACV IHD-J (biotinylation performed using a commercial kit: EZ-Link sulfo-NHS-biotin; Pierce-Thermo Scientific, USA) attached to streptavidin-coated magnetic beads (Dynabeads; Invitrogen, USA). All routine phage display techniques were performed essentially as described (67). Blocking of plates, beads, and phages was conducted for 60 min using two blocking solutions: 3% BSA (in PBS) or 2% skimmed milk and 0.05% Tween 20 in PBS. The blocking solutions were alternated between panning cycles. All washing steps were performed using PBST (PBS containing 0.05% Tween 20 and 2% BSA) or PBS. For each panning cycle, 1 × 10^7^ PFU/mL virus was used to coat the polystyrene plate, and after an overnight incubation, plates were washed and blocked. Biotinylated-VACV IHD-J was incubated with 100 µL streptavidin-coated magnetic beads for 30 min, followed by blocking. For the first panning cycle, approximately 1 × 10^11^ phages were incubated with the antigen-coated plates for 60 min or with the blocked beads for 90 min, followed by a total of six washes with PBST for the plates or beads. Phages were eluted by incubation with 1 mL of 100 mM triethylamine (Sigma, Israel) for 30 min, and following neutralization (in 200 µL 1 M Tris-HCl, pH 7.4), they were used to infect 5 mL of *E. coli* TG1 strain by incubation at 37°C for 30 min without shaking, followed by 30 min at 120 rpm. The bacterial culture was plated on YPD-Amp-Glu agar and incubated overnight at 30°C. Clones were harvested into 5 mL of YPD-Amp-Glu with a 20% glycerol solution, and phage production for the next round of panning was conducted in 10 mL of medium, as described above. Two additional panning cycles were conducted essentially similarly, with the following modifications: 10^10^ and 10^9^ phages were used as input (for the second and third cycles, respectively), and the washing steps were increased to include 10 washes of PBST for the antigen-coated plates and 10 washes (×8 PBST and ×2 PBS) for the beads.

Single colonies were randomly picked from the third cycle output, and a screen of specific binders was performed using phage ELISA against VACV IHD-J or ΔH3.

### Single chain Fv individual clone diversity and sequence verification

TAB-RI_For (CCATGATTACGCCAAGCTTTGGAGCC) and CBD-AS_Rev (GAATTCAACCTTCAAATTGCC) phagemid-specific primers were used for colony PCR and sequence analysis of scFv Ab individual clones. Colony PCR products were analyzed on a 1.5% agarose gel to confirm the intactness of the scFv. Restriction fragment size polymorphism was performed using *MvaI* (FastDigest #FD0554; Thermo Scientific, USA) to evaluate the sequence variability of scFv individual clones. Following colony PCR, 5 µL of the PCR products was taken directly for restriction with 0.5 µL *MvaI* and 1 µL buffer ×10 (provided by the manufacturer) in a 10-µL reaction volume. Restriction was conducted for 1 h at 37°C, and the entire reaction mix was then resolved on a 2.5% agarose gel. Nucleic acid sequence analysis of individual scFv fragments was performed on the colony PCR product using the SeqStudio Genetic Analyzer (Applied Biosystems, USA).

### Antibody profile analysis

Antibody profiling of serum samples was conducted on a protein microarray containing 224 proteins of the vaccinia virus by Antigen Discovery Inc. (Irvine, CA, USA). The VIG used for analysis had an IgG content of 50 mg/mL and was provided by Omrix Biopharmaceuticals Ltd. (Omr IgG-am 5% VIG) (68). VIG was used for qualitative comparison at the same dilution as the serum samples.

### Production of antibodies

Phagemid DNA of the desired clones was isolated using the QIAprep spin Miniprep kit (Qiagen, GmbH, Hilden, Germany), and the entire scFv was cloned into a pcDNA3.1+-based expression vector that was modified, providing the scFv with the human (IgG1) CH2-CH3 Fc fragments, resulting in the scFv-Fc antibody format. Antibodies were expressed using the ExpiCHO Expression System (Thermo Scientific, USA, Cat# A29133) and purified on the HiTrap Protein-A column (GE Healthcare, UK).

### ELISA

Direct ELISA (65) consisted of coating microtiter plates with 1 × 10^7^ PFU/mL of inactivated VACV IHD-J or ΔH3 or with 2 µg/mL recombinant proteins. For phage ELISA, HRP-conjugated anti-M13 antibody (Sino Biological, USA, Cat# 11973-MM05T-H lot HO13AU601; used at 250 ng/mL) was used following detection with TMB substrate (Millipore, USA). ELISA of both serum and recombinant antibodies was applied with AP-conjugated donkey anti-human IgG (Jackson ImmunoResearch, USA, Cat# 709-055-149 lot 130049; used at 1:2,000 working dilution) following detection using PNPP substrate (Sigma, Israel).

### Plaque reduction neutralization test

Plaque reduction neutralization test was conducted as previously described (69). Briefly, all serum samples were heat inactivated (at 56°C for 30 min) and then diluted in twofold serial dilutions (between 1:100 and 1:25,600). Monoclonal antibodies were serially diluted twofold, starting at 20 µg/mL. Diluted sera were incubated with 500 PFU/mL of the following VACV strains: Lister, Copenhagen-vFIRE, WR-vFIRE, IHD-J-vFIRE, Wyeth-vFIRE, or Mpox 2018/2022 (1 h at 37°C). Diluted antibodies were incubated with 500 PFU/mL of either Mpox 2018 or Mpox 2022 (1 h at 37°C). Vero cells were infected with the virus-serum or virus-antibody mixtures and incubated at 37°C and 5% CO_2_ for 1 h. Overlay was then applied, and the plates were incubated for an additional 72 h. Following incubation, the overlay was aspirated, and the cells were fixed and stained with 1 mL/well of crystal violet solution. Plaques were counted, and the NT_50_ value was calculated using GraphPad Prism 6 software (GraphPad Software Inc., San Diego, CA, USA). Each sample was tested once, with several internal controls to ensure the validity of the test.

### *In vitro* neutralization test

Vero cells were seeded at 3 × 10^4^ cells in 100 µL of DMEM supplemented with 10% FBS, 1% glutamine, 1% NEAAs, and 0.5% Pen Strep Nistatin in 96-well plates and incubated overnight at 37°C for 1 h in 5% CO_2_. Antibodies were serially diluted 2- or 10-fold starting from 20 µg/mL, added to 0.1 MOI vaccinia WR-vFIRE virus and 1% guinea pig complement sera, and incubated at 37°C for 1 h in 5% CO_2_. The antibody-virus mixtures were then transferred to 96-well plates containing the cell monolayers, and the plates were incubated for 24 h at 37°C and 5% CO_2_. Following incubation, the cells were washed once with PBS before lysis with 25 µL/well of cell culture ×5 lysis buffer reagent (Promega) and incubated for 15 min at room temperature. Luciferase assay substrate (25 µL per well) was added, and the luminescence was immediately measured using an infinite 200pro reader (Tecan). The neutralization value was calculated using the following equation:

Each antibody dilution was run in duplicate with appropriate positive and negative controls. Each antibody was tested at least 10 times. A VIG sample was used for qualitative comparison in the same serial dilutions, starting from 62.5 µg/mL.

### *In vitro* comet inhibition assay

BSC-1 cells were seeded in 12-well plates (3 × 10^5^ cells/well) and incubated overnight at 37°C. Cells were infected with 10 PFU/well of vaccinia IHD-J, and incubated at 37°C for 1.5 h. After washing with MEM containing 2% FBS, the cells were overlaid with 1 mL/well of the same medium supplemented with 1.5% carbonate bicarbonate (overlay). Antibodies were then added to the overlay at concentrations ranging from 2.5 to 20 µg/mL in triplicate. The cells were incubated for 2 d at 37°C and then fixed and stained with crystal violet. Each antibody was tested at least three times. A VIG sample was used for qualitative comparison at the same concentrations.

### Biolayer interferometry assays

Binding studies were carried out using the Octet system (ForteBio, USA, Version 8.1, 2015) that measures BLI. All steps were performed at 30°C with shaking at 1,500 rpm in a black 96-well plate containing 200 µL solution in each well. Nickel-coated biosensors (Ni-NTA) were loaded with his-tagged protein (50 nM) and then reacted with increasing concentrations of the relevant antibody. Binding and dissociation were measured as changes over time in light interference after subtraction of parallel measurements from unloaded biosensors. Sensorgrams were fitted with a 1:1 binding model using the Octet data analysis software 8.1 (Fortebio, USA, 2015).

For the binning experiments of antibody pairs, antigen-loaded sensors were incubated with a fixed concentration of the first antibody (mAb1; 10 µg/mL) until saturation was reached and then incubated with the second antibody (mAb2). In each set of experiments, the background signal was obtained from a parallel sensor incubated with the homologous antibody (mAb1), and sensograms are presented after subtraction of the background signal.

### Statistical analysis

For analysis of differences between *in vitro* neutralization results, area under the curve (AUC) was calculated for each curve, and one-way ANOVA and Tukey’s post-test were conducted to determine significance between all groups. For comparisons of two groups, a two-tailed, unpaired *t*-test was used. For comparison between groups in Fig. 2, a paired test was used. All calculations were performed using GraphPad Prism 5.
